# Assessment of population coverage of hypertension screening in Thailand based on the effective coverage framework

**DOI:** 10.1186/s12913-018-2996-y

**Published:** 2018-03-27

**Authors:** Kulpimol Charoendee, Jiruth Sriratanaban, Wichai Aekplakorn, Piya Hanvoravongchai

**Affiliations:** 1 Department of Preventive and Social Medicine, Faculty of Medicine, Chulalongkorn University, Bangkok, 10330 Thailand; 2 Department of Community Medicine, Faculty of Medicine, Ramathibodi Hospital, Mahidol University, Bangkok, 10400 Thailand

**Keywords:** Hypertension, Screening, NCDs, Measure, Effective coverage, Access, Thailand

## Abstract

**Background:**

Hypertension (HT) is a major risk factor, and accessible and effective HT screening services are necessary. The effective coverage framework is an assessment tool that can be used to assess health service performance by considering target population who need and receive quality service. The aim of this study is to measure effective coverage of hypertension screening services at the provincial level in Thailand.

**Methods:**

Over 40 million individual health service records in 2013 were acquired. Data on blood pressure measurement, risk assessment, HT diagnosis and follow up were analyzed. The effectiveness of the services was assessed based on a set of quality criteria for pre-HT, suspected HT, and confirmed HT cases. Effective coverage of HT services for all non-HT Thai population aged 15 or over was estimated for each province and for all Thailand.

**Results:**

Population coverage of HT screening is 54.6%, varying significantly across provinces. Among those screened, 28.9% were considered pre-HT, and another 6.0% were suspected HT cases. The average provincial effective coverage was at 49.9%. Around four-fifths (82.6%) of the pre-HT group received HT and Cardiovascular diseases (CVD) risk assessment. Among the suspected HT cases, less than half (38.0%) got a follow-up blood pressure measurement within 60 days from the screening date. Around 9.2% of the suspected cases were diagnosed as having HT, and only one-third of them (36.5%) received treatment within 6 months. Within this group, 21.8% obtained CVD risk assessment, and half of them had their blood pressure under control (50.8%) with less than 1 % (0.7%) of them managed to get the CVD risk reduced.

**Conclusions:**

Our findings suggest that hypertension screening coverage, post-screening service quality, and effective coverage of HT screening in Thailand were still low and they vary greatly across provinces. It is imperative that service coverage and its effectiveness are assessed, and both need improvement. Despite some limitations, measurement of effective coverage could be done with existing data, and it can serve as a useful tool for performance measurement of public health services.

## Background

Hypertension is a major health problem facing many countries around the world including Thailand. It is a major risk factor leading to illnesses and deaths from cardiovascular diseases [1]. Approximately one-third of the world’s population has high blood pressure, and the number tends to increase more rapidly. It was estimated that in 2025 the number of people with hypertension would rise by 60% to about 1.56 billion [2]. The Global Burden of Disease Study 2013 put hypertension as a major risk factor for premature deaths globally [3].

An earlier survey in Thailand found an increased prevalence of hypertension among those aged 15 years and over during the last 5 years from 21.4% to 24.7%. Among them, 40% were unaware of having hypertension [4]. The Thai Burden of Disease Study 2009 reported hypertension as the second and third risk factors for DALYs for male and female, respectively [5].

Prevention or delay of the new HT cases is a key strategy under the Global Action Plan for the Prevention and Control of Non-Communicable Diseases (NCDs) with the goal to reduce illnesses and premature deaths from cardiovascular diseases [6]. HT screening is thus an important initial step leading to further adequate management. Actual health benefits can be achieved when effective hypertension screening is supported by adequate and continuous care for those in need [7]. A previous study revealed that hypertension screening in population age 65 year or older contributed to reduced rates of annual hospitalization due to cardiovascular diseases [8]. Systematic reviews by the U.S. Preventive Services Task Force reported that substantial indirect evidence continues to support the net benefit of screening for high blood pressure in adults aged 18 years or older [9].

In Thailand, HT screening is a basic healthcare service that has been widely available with the screening mostly performed in the community. Previously, HT screening performance was measured only by assessing service coverage. However, earlier studies showed that high rate of service coverage did not necessarily imply good health outcome. A significant proportion of those found during screening to be at risk from the disease did not get adequate care or follow up treatment. Hence, it is important to take into consideration not only HT service coverage but also its screening quality and effectiveness [10].

Health system can contribute to health improvement of the people by means of delivering high-quality service to those who will benefit from them. It is therefore important for policymakers and those involved in health service system governance to have accurate information especially in relation to the existing service gaps, both in term of population coverage and its effectiveness. In 2003 the World Health Organization (WHO) introduced effective coverage “framework” [11, 12] as a tool for health system performance assessment. Effective coverage is defined as “the fraction of potential health gain that is actually delivered to the population through the health system.” It is a significant advancement over the usual approach of measuring crude coverage. The effective coverage framework proposes to measure three components, which include health need, utilization, and service effectiveness and aggregates them into a single metric. It can be used in an assessment of individual or population level, and it allows for tracking changes and benchmarking of the health system performance. Over the years, the concept of effective coverage has been adopted to measure and assess a wide range of health services [11, 13, 14]. The objective of this study is to measure the effective coverage of hypertension screening services at the provincial level in Thailand, using an applied effective coverage framework.

## Methods

This study focuses on the assessment of HT prevention coverage at the individual level using providers’ perspective. It applied the effective coverage framework to measure comprehensive HT screening services including after screening activities in three specific groups: those with pre-HT; suspected HT; and confirmed HT.

We obtained a secondary dataset from the administrative databases of the National Health Security Office (NHSO). This dataset derived from the outpatient service database for health promotion and disease prevention which covered over 40 million Thais in 76 provinces. NHSO collected data on individual health care service utilization at the outpatient department of health care providers under the Ministry of Public Health into 21 standard dataset files. We used 6 files from 2013 which contain information on population characteristics, HT screening, health care utilization, HT registry, HT patients follow up.

The study samples were all Thai population aged 15 years and older whose information was available in the NHSO database. We included all Thais who registered in the national civil registration system and lived in the 76 study provinces. Those having been previously diagnosed with HT were excluded because they were not considered in the HT screening target group.

Measurement of effective coverage of hypertension screening was based on the method originally proposed by Shengelia et al. It is based on the following formula [11, 12]:

$$ {EC}_{\mathrm{ij}}={Q}_{\mathrm{ij}}{U}_{\mathrm{ij}}\mid {N}_{\mathrm{ij}} $$

where Q is the quality, or health gain ratio, i.e., the gain provided to a person by intervention in relation to the maximum possible health gain; this study measured by the proportion of effectiveness of service in relation to the benefits received from the service;

U is the utilization of health service and refers to the probability that the individual with a need will receive the intervention; this study measured by the proportion of service received by an individual; and.

N is need indicator which refers to individuals who will gain actual benefits from receiving or true need; if *N* = 1 is the true need for receiving healthcare services, *N* = 0, then the individual does not have a need for coverage.

Our operational definition of the population in need of HT screening follows the official criteria for the target population of HT screening as identified by the Ministry of Public Health in 2013. It included all Thai individuals aged 15 and older who live in the 76 provinces outside Bangkok. Bangkok was not included in the study because it has its own service delivery system governance with a separate information management system.

Note that for Shengelia, quality is synonymous with effectiveness. In this study, we identified the effectiveness of HT screening based on a set of effectiveness criteria, which were constructed by the findings from a systematic literature review and a consultation with six national experts. The HT screening service in this study includes HT screening activities and subsequent post-screening health care needs such as risk assessment, disease detection, and monitoring to control blood pressure and prevention of complication from cardiovascular diseases.

The effectiveness criteria (Q) of HT screening depended on the quality and completeness of a set of activities provided to the target groups. They ranged from blood pressure screening for non-HT population, to delivery of interventions to reduce HT risks, and to blood pressure management among the population at risk and newly diagnosed HT cases. The quality or effectiveness criteria were set up to assess these population subgroups, classified at the time of the initial screening by their blood pressure level. The ranges were SBP < 120 and DBP < 80 mmHg for normotension, SBP 120–139 and/or DBP 80–89 mmHg for pre-HT, and SBP ≥ 140 and/or DBP ≥ 90 mmHg for suspected HT.

Table 1 shows the set of interventions and effectiveness criteria for these population subgroups. For example, in addition to blood pressure (BP) screening, anyone with BP in a pre-HT level must also obtain CVD risk assessment. Based on the measurements of effective coverage of each particular service at the individual level, aggregate summary statistics of effective coverage at the province and national levels were calculated.

**Table 1:** Criteria for measuring effective coverage of hypertension screening and prevention services

Target population subgroups	Intervention sets	Effectiveness criteria (Q)
Normotension	BP measurement	Receiving at least one BP measurement in a year
Pre HT	HT& CVD risk assessment^a^	Receiving HT & CVD risk assessment
Suspected HT	Diagnosis confirmation	Receiving repeated BP measurement within 2 months after initial screening
Newly diagnosed HT	CVD risk assessment^b^	Receiving CVD risk assessment
	Timely treatment	Receiving early treatment
	Controlling BP & risk factors	Blood pressure lower than the initial level or under control (Systolic BP < 140 mmHg and Diastolic BP < 90 mmHg) with serum lipid level better than the initial test.

^a^HT&CVD risk assessment for the pre-HT group includes history taking on age, sex, diabetic, smoking, and alcohol drinking history, and physical check-up for weight, height, waist circumference, and blood pressure measurement

^b^CVD risk assessment for newly diagnosed HT case includes blood test for total cholesterol, low-density lipoprotein, and high-density lipoprotein level

The study protocol was approved by the Institutional Review Board, Faculty of Medicine, Chulalongkorn University, Bangkok, Thailand on 4 April 2015 (Reference number 117/57). The 2013 dataset was used with the permission of NHSO.

## Results

Table 2 shows general characteristic for the 76 provinces in 2013. The information was gathered from various databases, mostly from national surveys and Ministry of Public Health statistics. There were huge differences between province. For example, the mid-year population in a province ranges from 0.2 to 2.6 million with an average number of 0.8 million.

**Table 2:** Characteristics for 76 provinces in Thailand in 2013 (*n* = 76)

	Mean	SD	Min	Max
Midyear population (million)	0.8	0.47	0.2	2.6
Age group (%)				
1–14 year	19.0	2.83	13.3	27.6
60 year and over	13.4	2.15	8.9	18.6
Education				
Literacy (%)	95.3	5.72	72.1	99.6
Economics				
Gross Provincial Product (100,000) per Capita	1.4	1.40	0.4	10.3
Living area (%)				
Urban	34.4	15.03	11.3	74.5
Public health resources				
Density of primary care unit per sq.km.	51.3	27.83	8.2	178.6
Population (1000) per primary care unit	6.1	2.74	3.3	17.9
Population (1000) per physician	4.2	1.41	1.8	12.0
Population (1000) per nurse	0.6	0.21	0.3	1.4
Population (1000) per public health personnel	1.6	0.57	0.7	3.8

**Fig. 1:**
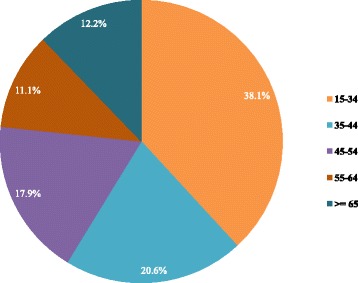
Age groups of Thai population 15 years old and over without established hypertension, 2013

**Fig. 2:**
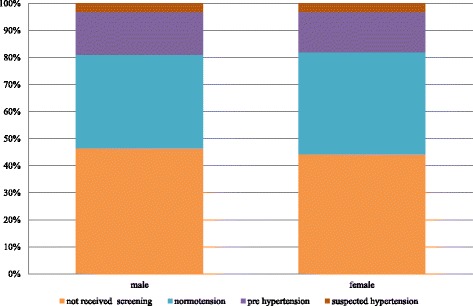
Proportion of Thai people with and without HT screening and proportion of HT screened individuals with normal blood pressure, pre HT, and suspected HT, by gender, 2013

**Fig. 3:**
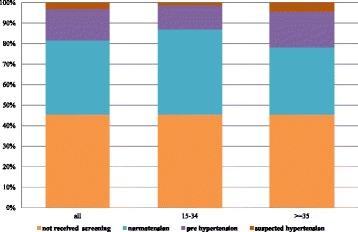
Proportion of Thai people with and without HT screening and proportion of HT screened individuals with normal blood pressure, pre HT, and suspected HT, by age group, 2013

There were approximately 42 million Thai population aged 15 or older without a prior diagnosis of hypertension, our target population for HT screening. There are equal numbers of males and females with almost 38% of them in the 15 to 34 years old age group (Fig. 1). A little over half of the target population (54.6%) have had an HT screening during the study period. The proportion getting screening is slightly bigger among female. Among those who received HT screening, the proportions of each population subgroup (normotension, pre-HT, or suspected HT) are quite similar for males and females (Fig. 2). Those aged between 15 and 34 years have a higher proportion of normotension while population aged 35 years and older have a higher proportion of suspected HT or pre-HT individuals (Fig. 3).

**Fig. 4:**
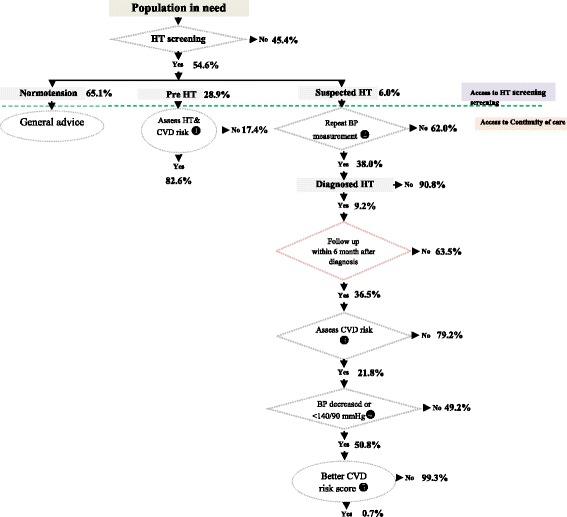
Pathway of HT screening and prevention and control service coverage, showing proportion of population in each subgroup and the services they received

The diagram in Fig. 4 shows the share of different subpopulation across all age group and the proportions of each subgroup who received HT prevention interventions. Around four-fifths (82.6%) of the pre-HT group received HT and CVD risk assessment. Among the suspected HT cases, less than half (38.0%) got a follow-up blood pressure measurement within 60 days from the HT screening date. Around 9.2% of the suspected cases were diagnosed as having HT, and only one-third of them (36.5%) received treatment within 6 months. In this confirmed group, 21.8% obtained CVD risk assessment, and half of them had their blood pressure under control (50.8%) with less than 1% (0.7%) of them managed to get the CVD risk reduced.

**Fig. 5:**
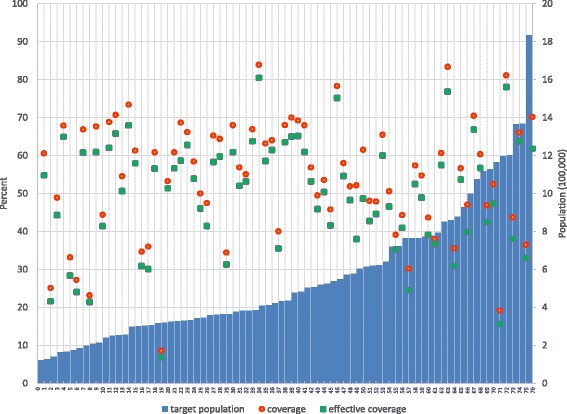
Distribution of coverage and effective coverage of HT screening per 100,000 population in each of the 76 provinces, ranked from lowest to highest number of target population in the province

**Fig. 6:**
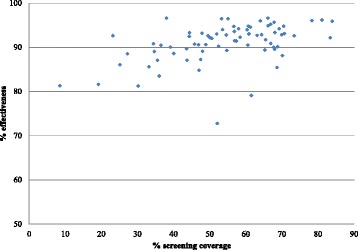
Distribution of effective coverage and coverage level in 76 provinces of Thailand in relation to key development and public health indicators in the year 2013

**Fig. 7:**
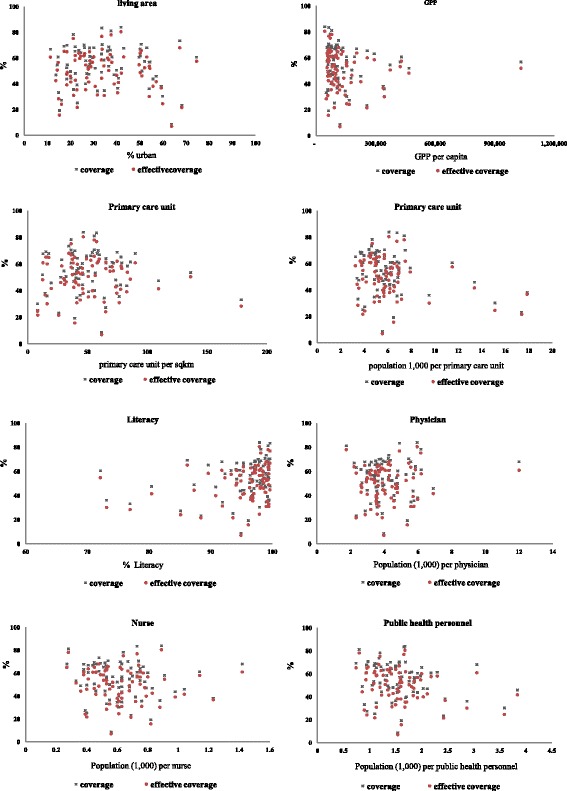
Two-way scatter plot between the percentage of HT screening coverage and proportion of prevention and control service effectiveness in each of 76 provinces

Considerable differences across provinces were observed. Our analyses revealed that the average provincial effective coverage of HT screening for the target population is at 49.9%. It varies greatly across provinces, from 6.9% to 80.5%. Effective coverage level was considerably lower than crude coverage for all provinces. The difference between effective coverage and coverage of HT screening also varies across provinces, from to 1.3% to 14.2%. The effective coverage at the province level correlates well with the population coverage measure, with a strong linear relationship between the two (*r* = 0.99, *p* < 0.001). There is no clear relationship between the effective coverage and the province size (population size) as shown in Fig. 5. However, there is a weak but significant correlation between the level of coverage of a province and its quality/effectiveness of the screening coverage for the population age 15 years and over as shown in Fig. 6. The results show no clear pattern in the distribution of coverage and effective coverage across key provincial statistics including the proportion of urban population, Gross Provincial Product (GPP), the density of primary care unit per area (square kilometer), the ratio of population to primary care unit, and the ratio of population to healthcare personnel as shown in Fig. 7.

## Discussion

Our study is the first implementation of the effective coverage framework proposed by the World Health Organization for health service assessment in Thailand. It measured a comprehensive set of services related to HT screening from pre-diagnosis interventions to treatment new diagnosed HT within the first six months. All of the components were assessed and summarized into a single metric.

The coverage of HT screening at 55% indicates that during the study period, just over half of the Thai population aged 15 years and above, who have never been diagnosed with hypertension before, received hypertension screening services. This is despite HT screening being a major health campaign by the government. This is not surprising. An earlier population sample survey also showed that around 40% of the hypertensive individuals were not aware that they are hypertensive [4]. The gap in screening coverage demonstrates that the service still does not reach all those in need which is a missed opportunity for disease prevention and control.

The barriers to service access could stem from any of the three dimensions as described in Evans et al. [15] namely, physical accessibility, financial affordability, and acceptability. In the Thai context, financial affordability should not be a major issue given HT screening is part of the benefit package of the Universal Health Coverage program that has been in place nationwide since 2002. Physical accessibility and acceptability could be more important. Even though most healthcare facilities offer outreach community HT screening services, the identified gap demonstrates that a significant proportion of the target population could not be reached.

Effective coverage of HT screening in Thailand (excluding Bangkok) was found to be only half of its full potential. A major portion of the effective coverage gap was due to incomplete screening coverage as discussed above. But there was also a big portion of those who were screened and considered as HT suspects but did not have access to a timely confirmation visit.

The variation in effective coverage of HT screening service across province deserves further deliberation. Huge difference in provincial level stats may indicate the differences in policies, procedures, and other factors. These include human resources (both quantity and quality), budget, technology and local environment. Existing literature shows that several factors can affect patient access and the decision of the patient to receive service. These include education level, awareness of information related to health and healthcare alternatives, location and distance of the service unit, convenience, travel cost and awareness of service quality [12, 16]. They can be classified into personnel factors (such as knowledge and skills, intention on reducing risk factors, social collaboration) and healthcare service system factors (including access, sufficiency and appropriateness of the service, acceptance in receiving service) [17]. In Thailand, both personnel factor and system factor could be key determinants. Further study is needed to understand these determinants to help improve the HT screening and related services in the country.

Our findings also reveal that there are relatively small differences between crude coverage and effective coverage statistics at the province level (range from 1.3% to 14.2% with an average difference of 4.2%). This can simply be interpreted as the gap in service effectiveness is small, and the gap in overall coverage is more important. However, there are three other possible explanations for such findings. First, the choice of the target population in Thailand to all people age 15 years and over resulted in a bigger population to screen and more of them in the normotension group. Both factors contributed to lower relative importance of the effectiveness component in the effective coverage assessment. Second, limited data availability in the secondary healthcare service dataset made it more difficult to assess service effectiveness. Third, the choice of effectiveness criteria used in this study where quality weight is set to either zero or one made the assessment not sensitive to the change in program effectiveness. Further studies are required to improve the tool as well as data availability for better assessment. Such improvement especially in the evaluation of NCDs service quality and effectiveness will provide additional insights into the performance of hypertension screening services in Thailand.

A number of limitations exist in this study. The measurement of comprehensive effectiveness was not possible with limited data. Hence, some components of quality or effectiveness measures were left out while others cannot be measured. The study relies on relatively concrete effectiveness or quality scores that may not fully reflect the potential benefit from obtaining a specific component of HT services. It requires further studies to assess the intervention effectiveness to fine tune the weights adhered to each quality or effectiveness component. The administrative data used in this study was limited by the completeness in itself and by the lack of data from health care providers outside the Ministry of Public Health, including the private sector. All these limitations should be overcome in further studies for the measurement to be more accurate.

A few recommendations are provided here for future studies. Determinants of provincial differences should be further investigated to identify relevant factors that influence HT screening and prevention coverage and their effective coverage. The tool for effective coverage measurement in HT screening can be refined with better design of effectiveness criteria. Future analyses may focus on specific groups such as the urban, non-urban, and vulnerable population groups. Nevertheless, our analyses show a significant gap in hypertension screening services in the country, which implies that improvement in primary care systems is required. For other health care interventions or services, effective coverage framework can be used to improve their performance assessment. It will be particularly useful for those programs that already have good service coverage to include the quality component in their assessment.

## Conclusion

Our findings suggest that hypertension screening coverage, post-screening service quality, and effective coverage of HT screening in Thailand were still low and they vary greatly across provinces. It is imperative that service coverage and its effectiveness are assessed, and both need improvement. Despite some limitations, the exercise in Thailand shows that the measurement of effective coverage could be done with existing data, and it can serve as a useful tool for performance measurement of public health services. The findings shed light for global universal health coverage monitoring effort where measurement of effective service coverage is a core component a health target of the Sustainable Development Goals.

## Abbreviations

BP: Blood pressure
CVD: Cardiovascular diseases
DALYs: Disability-Adjusted Life Years
GPP: Gross Provincial Product
HT: Hypertension
NHSO: National Health Security Office
NCDs: Noncommunicable Diseases
WHO: World Health Organization
